# The seed dispersal syndrome hypothesis in ungulate-dominated landscapes

**DOI:** 10.1038/s41598-024-55820-0

**Published:** 2024-03-05

**Authors:** Jose M. Fedriani, Pedro J. Garrote, Tamara Burgos, Gema Escribano-Ávila, Brayan Morera, Emilio Virgós

**Affiliations:** 1 Centro de Investigaciones Sobre Desertificación CIDE, CSIC-UVEG-GV, Carretera de Moncada a Náquera, km 4.5, 46113 Moncada (Valencia), Spain; 2 Estación Biológica de Doñana (EBD - CSIC), c/Americo Vespucio 26, 41092 Seville, Spain; 3 Área de Biodiversidad y Conservación, Departamento de Biología, Geología, Física y Química Inorgánica, Rey Juan Carlos University, Madrid, Spain; 4 Biodiversity, Ecology and Evolution Department, Biological Science Faculty, Universidad Complutense de Madrid, Ciudad Universitaria, C/ José Antonio Novais 12, Madrid, Spain

**Keywords:** Community ecology, Ecology

## Abstract

The Seed Dispersal Syndrome Hypothesis (SDSH) posits that fruit traits predict the main dispersers interacting with plant species. Mammalian dispersers, relying heavily on olfactory cues, are expected to select dull-colored, scented, and larger fruits compared to birds. However, challenges like overabundant seed predators and context-dependency of frugivore-plant interactions complicate SDSH expectations. We studied the Iberian pear, *Pyrus bourgaeana*, an expected mammal-dispersed tree based on its fruit traits. Extensive camera-trapping data (over 35,000 records) from several tree populations and years revealed visits from seven frugivore groups, with ungulate fruit predators (59–97%) and carnivore seed dispersers (1–20%) most frequent, while birds, lagomorphs, and rodents were infrequent (0–10%). Red deer and wild boar were also the main fruit removers in all sites and years but acted as fruit and seed predators, and thus likely exert conflicting selection pressures to those exerted by seed dispersers. Although, as predicted by the SDSH, most Iberian pear fruits were consumed by large and medium-sized mammals, the traits of Iberian pear fruits likely reflect selection pressures from dispersal vectors in past times. Our results do not challenge the SDHS but do reveal the importance of considering frugivore functional roles for its adequate evaluation.

## Introduction

Fruit-frugivore interactions are critical for the dynamics, evolution, and conservation of plant communities worldwide^1–4^. Fruits provide frugivores with nutrients and water essential for their reproductive success and survival^4^. Plants benefit from the activity of certain frugivores by having their seeds dispersed which increases both local recruitment (i.e., short-distance dispersal) and also gene flow and the (re)colonization of vacant habitats (i.e., long-distance dispersal;^5–8^. Since fruit traits can influence foraging choices by mutualistic seed dispersers and, consequently, the probability of seed dispersal, specific combinations of fruit traits (e.g., color and size) that favor interactions with particular seed dispersers are known as seed dispersal syndromes^9–12^. Under the Seed Dispersal Syndrome Hypothesis (SDSH), fruit traits can predict the main type of dispersers with which plant species interact. For instance, avian seed dispersers are predicted to select brightly colored (red, blue and black) and small fleshy fruits because birds have acute color vision and commonly swallow fruits whole. Mammalian seed dispersers should select dull-colored (brown and dark green), scented and larger fruits, on average, compared to the bird syndrome, as mammals do not rely heavily on visual cues to find fruits and can eat fruits piecemeal^10,11,13–15^. Nonetheless, it is important to correctly differentiate between the set of traits that favor a particular dispersal mechanism (i.e., seed dispersal syndrome) and the vectors that move the seeds in a particular location and time (i.e., actual seed dispersal). Therefore, the presence of any seed dispersal syndrome does not preclude some seed dispersal by mechanisms other than the predicted one^16^.

Though the SDSH has regained attention and has been valuable in understanding plant-frugivore interactions^12,16–18^ some important drawbacks persist. Firstly, certain fruit traits (color, size) associated with seed dispersal syndromes are more influenced by shared evolutionary history among plant taxa than by direct selection pressures exerted by dispersers (i.e. phylogenetic constraints;^19,20^). Secondly, while such fruit traits may attract mutualistic seed dispersers, they may also attract antagonistic frugivores that act as seed predators or pulp feeders^21^. Antagonistic frugivores can exert selection pressures in opposite direction to that exerted by seed dispersers and thus, disrupt the predicted correlations between fruit traits and seed dispersers^22–25^. Thirdly, the variability and context-dependency of frugivore-plant interactions^26,27^ also pose significant challenges to the SDSH. Frugivore assemblages and their seed dispersal effectiveness can vary across different habitats, regions, and seasons even for a given disperser species^6,27–29^, potentially altering patterns of frugivory and thus the selective pressures expected from dispersal syndromes. Therefore, a comprehensive evaluation of the SDSH requires studies that account for the functional role of frugivores (e.g., seed dispersers *vs*. seed predators) and are replicated across sites and fruiting seasons.

Furthermore, during the last few decades some management practices such as those related to hunting can extremely alter frugivore assemblages. In many human-altered areas, limiting culling policies, food supplementation, and predator removal often led to overabundant ungulate populations^30–33^. These increased populations can alter ecosystem functioning in general and plant-seed disperser interactions in particular^33–35^. Ungulates feed intensively on ripe fruits and can act as either seed/fruit predators^21^ or seed dispersers^36^, depending on a combination of plant (e.g., fruit size and shape, seed toughness) and animal traits (e.g., foraging behavior, body size, gut characteristics;^27,37^). When acting as seed predators, overabundant ungulates have the potential to limit the number of fruits to dispersers and also to exert conflicting and overwhelming selection pressures on fruit traits to those exerted by seed dispersers^23^. Furthermore, by limiting the number of available fruits, ungulates could influence in the feeding decisions of other frugivores making them less choosy^38,39^. Surprisingly, despite the marked increase of ungulate populations in many ecosystems^34,40^, the implications of such novel selection pressures have been largely neglected in evaluations of the SDSH.

In this study, we illustrate the importance of accounting for the functional role of frugivores (e.g., seed dispersers *vs*. seed predators) in assessing the SDSH. To this end, we focus on the interaction between the Iberian pear, *Pyrus bourgaeana* Decne (Rosaceae), and its vertebrate fruit consumers in various Mediterranean ecosystems marked by overabundant ungulate populations in southern Spain. The fruits of this tree are notable for their size (2–4 cm in diameter), brownish hue, and potent aroma when ripe. As anticipated by the SDSH^13,41,42^, numerous mammal species consistently harvest a significant portion of the tree's fruit yield^29,43,44^. For instance, medium-sized carnivores typically consume the entire fruits, internally transporting the seeds and facilitating their dispersal, while wild ungulates generally ingest whole fruits, grinding most of the seeds they consume, acting thus as seed predators^45^. Despite prior research on the dispersal ecology of the Iberian pear, there still exists a knowledge gap concerning the consistency of the role of seed dispersers and seed predators over both space and time and the implications of this within the SDSH. Additionally, limited information is available regarding the contribution of smaller fruit consumers such as birds, lagomorphs, and rodents^23^. To address this gap in our understanding, we integrated a comprehensive dataset that includes both published and unpublished camera-trap observations of frugivore activity across various Iberian pear populations and years. Specifically, we aimed to (*i*) evaluate the spatial and temporal consistency in the relative importance of different functional groups of frugivores, and (*ii*) evaluate the prediction derived from the SDSH, that, in most locations and years, seed-disperser mammals (i.e. carnivores) rather than fruit and seed predators (i.e. ungulates) would dominate as the primary consumers of Iberian pear fruits.

## Results

### Relative importance of frugivore functional groups

**Figure 1:**
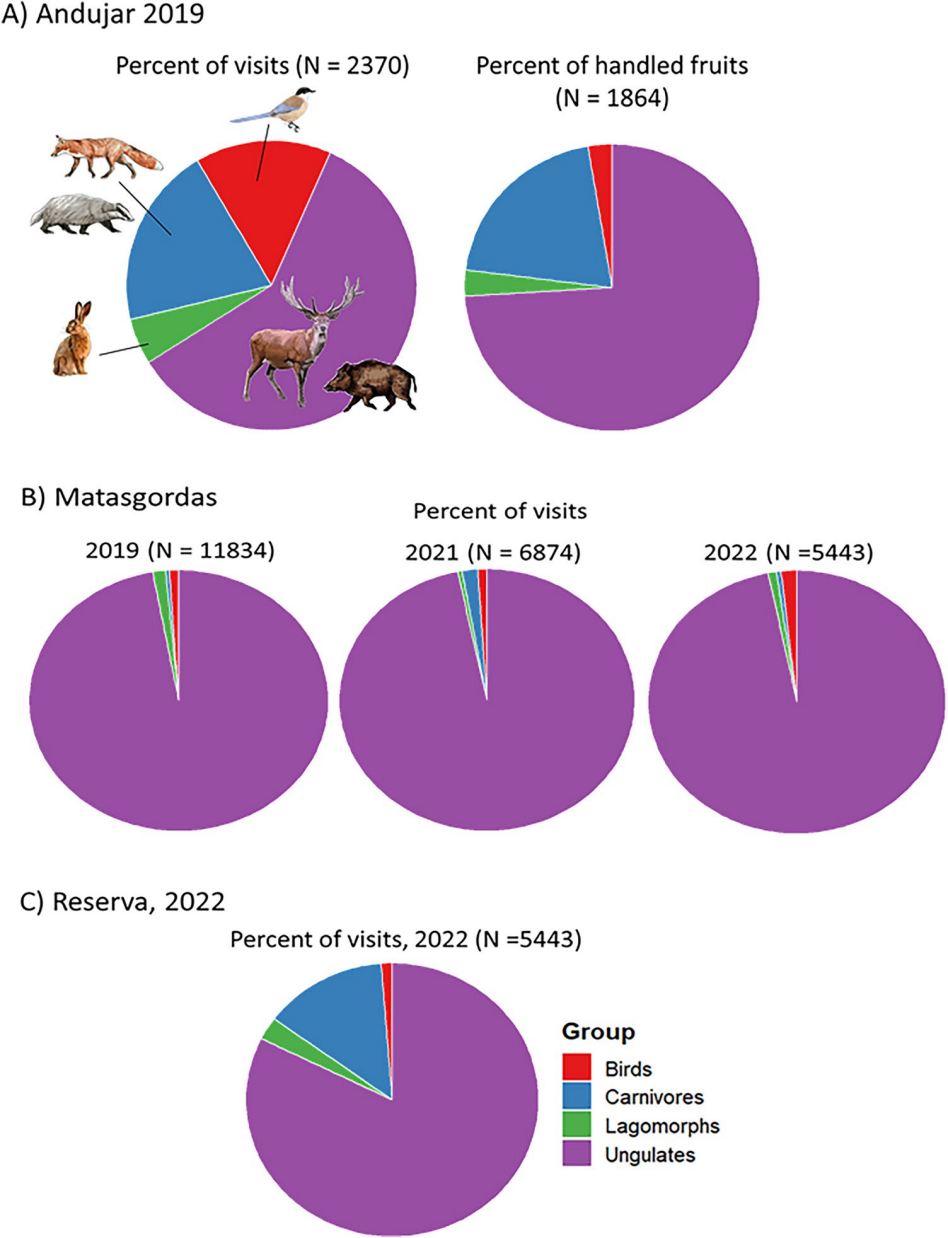
Frequency of visits and number of visited *Pyrus bourgaeana* trees by the main four functional groups in the three study sites (Andujar, Matasgordas, Reserva) within southern Spain. The Andujar and Reserva populations were sampled during the fruiting seasons of 2019 and 2022, respectively. The Matasgordas populations was sampled during 2019, 2021 and 2022 fruiting seasons. The percent of handled fruits by the main frugivore functional groups in Andujar are also shown (A).

Overall, fruiting trees were visited by the seven functional groups comprising 32 vertebrate species (Table 1). The most frequent visitor were always ungulates (59.2–97.1%) and, in particular, red deer (56.5–83.4%) and wild boar (1.9–30%; Fig. 1). Furthermore, ungulates were very reliable visitors, with either red deer (Andujar) or red deer plus wild boar (Matasgordas and Reserva) visiting all target fruiting trees.

**Table 1:** Frequency of visits and number of visited tree by different frugivore species in the three study sites within southern Spain.

		Andujar				Matasgordas				Reserva	
		2019		2019		2021		2022		2022	
		% visits	# visited trees	% visits	# visited trees	% visits	# visited trees	% visits	# visited trees	% visits	# visited trees
	Role	(n = 2370)^a^	(n = 30)	(n = 11,834)	(n = 12)	(n = 6873)	(n = 12)	(n = 5443)	(n = 9)	(n = 8829)	(n = 11)
Ungulates		**59.2**	**30**	**97.1**	12	**96.5**	**12**	**96.6**	**9**	**82.3**	**11**
Red deer	SP	56.5	30	83.4	12	68.2	12	66.3	9	58.3	11
Fallow deer	SP	0.7	6	0.1	4	0.1	4	0.2	4	0.3	4
Wild boar	SP/LD	1.9	21	13.6	12	28.2	12	30.0	9	23.8	11
Pyrenean ibex	SP	< 0.1	1	0.0		0.0		0.0		0.0	
Livestock		**0.0**		**0.0**		**0.1**		**0.1**	**1**	**2.7**	**9**
Cow	SP/LD	0.0		0.0		0.0		0.0		0.8	9
Horse	SP/LD	0.0		0.0		0.1		0.1	1	1.9	7
Carnivores		**20.2**	**29**	**0.4**	**8**	**1.7**	**11**	**0.5**	**7**	**13.7**	**11**
Red fox	LD	17.3	29	0.1	5	0.5	8	< 0.1	2	12.9	11
Eurasian badger	LD	2.0	12	0.3	8	1.1	11	0.5	5	0.5	10
Beech marten	LD	0.6	4	0.0		0.0		0.0		0.0	
Genet	LD	0.0		0.0		0.0		0.0		0.4	4
Egyptian mongoose	LD	0.3	3	0.0		0.1	4	< 0.1	2	< 0.1	2
Lagomorphs		**5.1**	**12**	**1.4**	**6**	**0.5**	8	**0.9**	**4**	**0.0**	**1**
European rabbit	PF	0.8	7	1.4	6	0.5	8	0.9	4	< 0.1	1
Granada hare	PF	4.3	6	0.0		0.0		0.0		0.0	
Rodents		**0.1**	**3**	**0.0**	**0**	**0.0**	**0**	**0.1**	**1**	**0.0**	
Wood mouse	SP	< 0.1	3	0.0		0.0		0.0		0.0	
*Rattus* spp.	SP	0.0		0.0		0.0		0.1	1	0.0	
Garden doormouse	SP	< 0.1	1	0.0		0.0		0.0		0.0	
Mediterranean pine vole	SP	< 0.1	1	0.0		0.0		0.0		0.0	
Corvids		**9.7**	**28**	**0.9**	**10**	**0.9**	**8**	**1.5**	**5**	**0.9**	**8**
Iberian magpie	PF/LD	9.7	28	0.8	10	0.8	8	1.3	5	0.0	
Eurasian magpie	PF/LD	0.0		0.1	1	0.1	2	0.1	2	0.9	8
Jaybird	PF/LD	< 0.1	1	0.0	0	0.0		0.0		0.0	
Other birds		**5.4**	**29**	**0.1**	**5**	**0.2**	**5**	**0.2**	**5**	**0.3**	**10**
European starling	PF/LD	0.3	7	0.1	3	0.1	1	0.0		0.3	4
Woodchat shrike	PF/LD	0.0		< 0.1	1	0.0		0.0		0.0	
Mistle thrush	PF/LD	1.4	15	< 0.1	1	0.0		0.0		< 0.1	1
Song thrush	PF/LD	0.6	7	0.0		0.1		0.0		0.0	
Wood pigeon	PF/LD	< 0.1	1	0.0		0.0	3	< 0.1	1	0.0	
Eurasian collared dove	PF/LD	0.1	1	0.0		0.0		0.0		0.0	
Black bird	PF/LD	2.8	10	0.0		0.0		< 0.1	2	< 0.1	1
Red-legged partridge	PF	0.2	3	< 0.1	1	< 0.1	1	0.2	5	< 0.1	2
European robin	PF	0.0		< 0.1	1	< 0.1	1	< 0.1	1	< 0.1	1
Common chaffinch	PF	0.0		0.0		0.0		< 0.1	1	0.0	
Great tit	PF	0.0		0.0		0.0		0.0		< 0.1	1
Black redstart	PF	0.0		0.0		0.0		0.0		< 0.1	2
Bluethroat	PF	0.0		0.0		0.0		0.0		< 0.1	2
European stonechat	PF	0.0		0.0		0.0		0.0		< 0.1	1

SP = Seed Predator; LP = Legitimate Disperser; PF = Pulp Feeder.

^a^It includes also three visits by non-frugivore vertebrates and one unidentified frugivore.

Despite the pervasive importance of ungulates as visitors, we found significant differences in the proportion of visits by different frugivore groups between study sites pairs (Chi-squared tests, χ^2^ ≥ 20.3, d.f. = 3, *P* < 0.001; Fig. 1). Such differences were even larger when we only compared pairs of study sites monitored during the same fruiting season (Andujar-Matasgordas 2019: χ^2^ = 3661.7, d.f. = 3, *P* < 0.001; Matasgordas-Reserva 2022: χ^2^ = 806.1, d.f. = 3, *P* < 0.001). Carnivores most frequently visited fruiting trees in Andujar (20.2%) and Reserva (15.1%) as compared to Matasgordas (0.4–1.7%; Table 1). Birds (especially corvids) were much more frequent visitors in Andujar (15.1%) than in Matasgordas and Reserva (always < 1.7%). Whereas corvids were relatively reliable visitors, other bird species visited only small subsets of target fruiting trees (Table 1). Lagomorphs were more frequent in Andujar (5.1%) than in Matasgordas and Reserva (0.0–1.4%). Rodents were very infrequent visitors in all study sites (≤ 0.1%; Table 1). Interannual differences only could be tested in Matasgordas. Though the relative frequency of visitation by different groups of frugivores varied annually (χ^2^ = 143.5, d.f. = 3, *P* < 0.001), ungulates in Matasgordas accounted for the vast majority of frugivore visits in the three sampled fruiting seasons (≥ 96.5%; Fig. 1B).

### Fruit removal and pulp feeding

In Andujar, frugivores were recorded handling about 70% of offered fruits (1887 out of 2700). Ungulates (mostly red deer) were by far the main fruit consumers, removing 1380 out of 1887 removed fruits (73.1%). Although they removed fruits in only 16.6% (n_total_ = 1404 ungulate visits) of their visits, those visits in which ungulates consumed the fruits they removed a large number of fruits (5.9 ± 7.9; mean ± 1SD). Carnivores were the second most important fruit removers in Andujar, accounting for 20.1% of total removed fruits (380 out of 1887; Fig. 1A). Most fruits removed by carnivores were due to red foxes (90.8%), followed by Eurasian badgers (8.2%) and then by beech martens (1.0%). Almost a third (142 out of 479) of carnivore visits resulted on fruit removal. On average, carnivores removed 2.7 ± 2.8 fruits per visit. Birds (mostly Iberian magpie and black bird) accounted for only 3.8% of handled fruits (i.e. 72 out of 1887). Such a low number of handled fruits was the result of a small fraction (10.6%) of visits leading to fruit handling (only 38 out of 360) and also of a low number of fruits handled per visit (1.3 ± 0.5). Furthermore, almost a third (23 out of 72) of fruits handled by birds consisted on fruit picking, not removal. Lagomorphs (mostly Iberian hare, but also European rabbit) only harvested about 3% (55 out of 1887) of total removed fruits (Fig. 1A). No rodent fruit removal was recorded.

Though fruit removal was not systematically quantified in Matasgordas and Reserva, our casual camera-trap observations indicated that ungulates were also the main fruit removers in these two tree populations. Specifically, we recorded 83 and 15 instances of fruit removal by red deer in Matasgordas and Reserva, respectively. Fruit removal by wild boar was recorded in five and three instances in Matasgordas and Reserva, respectively. Red foxes in Matasgordas were recorded removing two fruits. In Reserva red foxes were recorded 54 times interacting with fruits, 12 times removing fruit plus 42 times climbing fruiting pear trees. Further, genets in Reserva were photographed 14 times climbing fruiting trees probably in search of fruit. Badger fruit removal was recorded only once in Matasgordas. Iberian magpies in Matasgordas were recorded in 43 instances harvesting fruit, whereas in Reserva Eurasian magpies and European starling were recorded in only seven and one instances, respectively. Furthermore, a large fraction (39.2%, n = 51) of fruit harvesting by birds in Matasgordas and Reserva corresponded to fruit picking rather than fruit removal. No fruit removal by lagomorphs or rodents was recorded in these two populations.

## Discussion

Understanding the functional diversity of frugivores and the temporal and spatial variations in the strength of their interactions with fruiting plants is crucial for assessing their potential impact on plant dynamics, potential selection pressures on fruit traits, and also to assess seed dispersal syndromes. To this end, we conducted an ample monitoring of a frugivore assemblage over several years and Iberian pear populations. As predicted by the SDSH, the majority of fruits were consumed by mammals. Intriguingly, however, most fruits were removed by red deer and wild boar that grind most ingested seeds acting thus as fruit and seed predators. These antagonistic fruit predators are likely limiting the availability of fruits to seed dispersers and potentially imposing selection pressures on fruit traits that run counter to those exerted by mutualistic seed dispersers. For example, Fedriani and Delibes^23^ showed throughout field experiments that both seed dispersers (i.e. carnivores) and fruit predators (i.e., ungulates) preferred large fruits and thus, that they exerted selection pressures on fruit size in opposite directions. Therefore, we propose that ungulate populations, which often act as seed predators and have become overabundant in recent decades^31,33,46^, are likely disrupting the selection scenarios exerted by seed dispersers in many fleshy-fruited plant communities.

### Antagonistic overabundant ungulates as main fruit consumers

Though ungulates were by far the most frequent frugivore visitors in all study sites and years (Fig. 1), their proportion of visits was larger in the two Doñana populations than in Andujar. These differences could relate to differences between study sites in ungulate and/or other frugivore densities^43^. Another non-exclusive possibility is that ungulates in Andujar are less prone to visit some fruiting pear trees due to their behavioral responses to hunting^47^ and/or predation risk by Iberian lynx^48^. Importantly, by lessening tree fitness and limiting the number of seeds that would be dispersed by carnivores, ungulates likely reduced the chance for selection by these mutualistic agents. Besides this, although ungulates visited most individual trees, the rates of fruit predation by ungulates varied enormously among trees. If this variation was based on heritable traits, predation pressure exerted by ungulates on populations of Iberian pear would affect the relative representation of different genotypes in future generations, having thus an additional impact on the selective processes among carnivores and the Iberian pear. Further research is certainly needed to evaluate the potential role of ungulates as selective agents.

The observed predominance of ungulates as Iberian pear fruit consumers likely occurs also in other regions within the tree distribution range (southern Iberian Peninsula and northern Morocco;^49^), where ungulate population densities are unnaturally high. Such high ungulate densities relate to recent extinctions and population declines of natural predators (i.e., the gray wolf;^50,51^) and human-managed factors related to hunting practices (e.g., supplemental food, introductions, etc.;^30,31,52^). Additionally, it is probable that overabundant ungulates play a significant role as fruit and seed predator for many other fleshy fruited^36,53,54^ and non-fleshy fruited species^55–57^ across the Iberian Peninsula and elsewhere. Thus, given the global trend towards increasing ungulate populations^31,33,46^, further research is essential to assess ungulate roles on plant dynamics (herbivory, seed predation, seed dispersal, physical and chemical engineering;^36,58^).

### The contrasting roles of carnivores and smaller frugivores

As reported elsewhere for other large-fruited species^59–62^, our study unequivocally confirms the importance of medium-sized carnivores, particularly red foxes and badgers (much less than foxes), as seed dispersers of the Iberian pear. This pattern was most evident in Andujar and Reserva populations, where foxes removed significant proportions of the available fruits. In Matasgordas, however, fox occurred in much lower frequencies likely attributable to a sharp decline in their population due to human activities (e.g., illegal poaching, road accidents, habitat use changes;^63^) and perhaps also to interspecific killing by the locally abundant Iberian lynx^29^. Although badgers in Matasgordas may partly compensate for the lack of fox seed dispersal, they are unlikely to fully replace them due to interspecific carnivore differences in patterns of habitat use and fecal marking behavior^63^. Seed dispersal limitation in Matasgordas likely contributes to the low local Iberian pear recruitment and adult aggregated pattern observed^8^. Indeed, field studies have indicated that this population exhibits very limited reproductive and regenerative capacity, featuring a skewed demographic structure with the majority of individuals in older age classes, few juveniles, and even fewer seedlings and saplings^64^. Nonetheless, other factors, such as the overabundance of seedling predators (i.e., ungulates) and summer droughts, likely also contribute to this situation^8^.

Our results also indicate that corvids may serve as alternative seed dispersers^44^ in areas where carnivores are scarce, such as Matasgordas (Fig. 1B). Corvids can act as seed dispersers either by consuming small pears entirely or, if they cannot swallow them whole, by carrying them in their beaks to preferred perches, where they may land and peck at them or drop them in flight (i.e. stomatochory;^65^). Further, corvids can consume both fallen fruits from the ground and also can feed on fruits directly from trees before falling (Authors *pers. obs.*), which could increase somewhat their importance as seed dispersers. However, corvids generally disperse seeds over much shorter distances^66–68^ than do carnivores^8,59^. Other smaller bird species visited fruiting trees, although they primarily acted as pulp feeders and seldom removed any fruits. Rabbits in Doñana act mostly as pulp feeders^23^, whereas hares in Andujar appeared to behave as seed predators (i.e., no remnants of fruits handled by hares, other than pedicels, were observed in 53 recorded instances). Pulp feeding by small birds and rabbits has been proven to increase seedling emergence (via disinhibition effect) and survival and thus, local tree recruitment^69^. Although our camera-traps provided limited information on the role of rodents, previous seed predation experiments conducted in Doñana^70^ and Andujar^71^ indicated that they primarily act as seed predators. However, rodents have also been reported to hoard Iberian pear fruits^63^, thereby playing a dual role as both predators and short-distance dispersers of Iberian pear seeds. Thus, it is unlikely that birds, lagomorphs or rodents will significantly compensate for the loss of seed dispersal services provided by carnivores.

## Concluding remarks

Ungulate fruit predators, rather than carnivore seed dispersers, overwhelmingly dominate as the primary consumers of Iberian pear fruits. Thus, the fruits of this species, characterized by dull colors and scented aroma, and large size, could not be explained based on current dominant selection pressures. Instead, these traits are likely vestiges selected by extant and extinct frugivorous fauna in past times where ungulates fruit predators were not as abundant as nowadays^33^. These results do not challenge the SDHS as it refers for the presence of evolutionary plant traits rather than actual ecological processes. Our results, however, do emphasize the importance of accounting for the functional role of frugivores when assessing the SDSH. Indeed, these plant traits could have evolved before the arrival of the *Pyrus* genus in the Iberian Peninsula, originating in Central Asia, as proposed for the ancestors of many present-day plants, including the sweet apple *Malus* pumila^72–74^. Specifically, large fruits in the Rosaceae appear to have evolved by hybridization and then by extinct megafauna seed dispersers^74^. Thus, current ecological conditions are likely significantly different from those under which the tree evolved in the western Mediterranean basin^75^. For instance, although ungulates (e.g. red deer, wild boar) have inhabited in the Doñana area, at least, for thousands of years^75,76^, their densities during the last few decades has increased dramatically^77^. Also, during the last millennium, at least two potential seed dispersers, the wolf *Canis lupus*^78^ and the brown bear *Ursus arctos*^79^, became extinct in Doñana, and further extinctions occurred during the Pleistocene (e.g., the Barbary macaque *Macaca sylvanis*;^80^). Such decline in seed disperser populations has been aggravated over the last few decades by a marked rise in of ungulate fruit predators. To conclude, our results indicates that the composition of the frugivore assemblage of the Iberian pear has dramatically changed in recent times due to global change drivers (e.g., defaunation, overfaunation) which likely alter the selective pressures acting on fruit traits. We highlight the importance of discriminating between seed dispersal syndromes (i.e. sets of traits that favor a particular mechanism) and the current assemblages of seed dispersers and predators in humanized landscapes. A rigorous evaluation of SDSH requires to account for not only the size and sensorial abilities of frugivore species or groups, but also for their functional roles (seed dispersers, seed predators, pulp feeders, etc.).

## Study sites and methods

### Study sites

Our study was conducted at three sites located in Southern Spain, one within Sierra de Andújar Natural Park (hereafter, Andujar; 38° 14′ N, 4° 4′ W, ~ 740 km^2^; altitude, 400–800 m) and two within the Doñana National Park (hereafter Matasgordas and Reserva; 37° 9′ N, 6° 26′ W, ~ 543 km^2^, altitude 0–40 m). All three sites have typical Mediterranean climates with high annual average temperatures (~ 18 °C) and limited rainfalls (500–700 mm): (i) Andujar is located within extensive private lands which vegetation is dominated by Mediterranean scrubland and holm oaks *Quercus ilex*. Some of the most common fleshy-fruited species are the strawberry tree *Arbutus unedo*, the mastic *Pistacea lentiscus* and the Iberian pear, (ii) Matasgordas is located in the northern portion of the Doñana. The most representative habitats in this site include a Mediterranean scrubland dominated by *P. lentiscus*, *Halimium* halimifolium, *Chamaerops humilis* and small clusters of trees species such as *Quercus suber*, Iberian pear, and *Fraxinus angustifolia*, and (iii) Reserva is located at the Doñana core and its main habitats comprise a scrubland dominated by *H. halimifolium* and *Stauracanthus genistoides*, with several fleshy-fruited species including *Rubus ulmifolius*, *C. humilis*, and Iberian pear. *Quercus suber* trees are scattered across the area.

### Study species

The Iberian pear is a small tree endemic to the Iberian Peninsula and North Africa^49^. Its distribution is highly fragmented, with trees occupying patches of Mediterranean scrubland at low densities (usually < 1 individual ha^−1^), with occupied patches isolated from each other by natural and human barriers^81^. The reproduction and regeneration of this tree is very limited due to factors such as high mortality of seeds and seedlings, and seed dispersal limitation^8^. Each tree produces yearly between ~ 200–1500 fruits. Fruits are globose, fleshy pomes (~ 9.5 g and 2—4 cm in diameter;^43^), with strong aroma when ripe. Each fruit includes 1–5 viable seeds (46–91 mg each;^45^) with easily breakable coats. After ripening, fruits drop to the ground from September to December and are harvested by a diverse coterie of mammalian and avian frugivores^29,43,44^.

The most common frugivores of the Iberian pear are: (i) wild ungulates such as red deer *Cervus elaphus* and wild boar *Sus scrofa* occur in high densities in both areas because lack of natural predators (i.e. the gray wolf *Canis lupus*) and, in Andujar, also because supplemental food by hunters*;* (ii) medium-sized carnivores such as the red fox *Vulpes Vulpes*, Eurasian badger *Meles meles*, and the common genet *Genetta genetta* are common in both areas, whereas the stone marten *Martes foina* occurs only in Andujar*;* (iii) corvids (magpie *Pica pica*, Iberian magpie *Cyanopica cooki* and, only in Andujar, the Eurasian jay *Garrulus glandarius*) and several passerine species; (iv) lagomorphs (Iberian hare *Lepus granatensis* and European rabbit *Oryctolagus cunniculus*); and (v) rodents (e.g., *Apodemus sylvaticus*, *Mus spretus*, *Rattus spp.*). Fedriani and Delibes^45^ provided quantitative data indicating that, in Doñana, carnivores act as seed dispersers of the Iberian pear whereas wild ungulates as fruit and seed predators. Also, rodents have been shown to act mostly act as seed predators both in Doñana^70^ and Andujar^71^. Very little information is available, however, concerning the role of lagomorphs and birds as either pulp feeders, seed predators or seed dispersers of the Iberian pear.

### Methods

To estimate the importance of different frugivores for the Iberian pear in Andujar, we used published data from Burgos et. al.^29^ as well as our own unpublished data. We considered 30 fruiting Iberian pear trees during the dispersal season of 2019 (from October 15th to October 30th) and placed fruit depots beneath each tree. Each fruit depot was comprised of 30 ripe fruits within a 1 m side squared plot. Fruits were set regularly in six lines (five fruits per line) about 10 cm apart. Overall, 2,700 Iberian pear fruits were offered in the 30 studied trees. This fruit arrangement allowed to systematically quantify the number of fruits consumed by each frugivore visitor by comparing every image with the previous one to count the number of fruits left. Also, we recorded frugivore visitation as the presence in the pictures of any vertebrate frugivore within or in the immediacy (i.e. ≤ 2 m) of our experimental fruit depots (i.e. irrespective of whether they feed on fruits). A camera trap was placed (Scoutguard SG562-C; white led) on each target pear tree trunk at the height of 60 cm with a slope of 45 degrees. We programmed the cameras to record three images per second when movement was detected, with a minimum time delay (0 s) between consecutive records to maximize the number of images taken per visit. Only visits separated by a period greater than 30 min or clearly identified as different individuals were considered. Fruit removal was recorded during 15 consecutive days. Camera traps were visited every 5 days to refill the fruit depots. In overall, cameras were working 437 trap-days (see^29^ for further details).

To estimate the importance of different frugivores in Matasgordas, we collected data for 12, 12 and 9 fruiting Iberian pears in 2019 (from September 19th to November 24th), 2021 (from September 22nd to November 24th) and 2022 (from September 26th to December 8th), respectively (^44^, Authors *unpublished data*). Also, in Reserva we monitored 11 fruiting trees during 2022 (from September 21st to December 13th; Authors *unpublished data*). In both study sites, camera traps (LTL ACORN 5310A, detection range = 18 m) were installed to collect data regarding to frugivore species that visited ripe fruiting trees, but we did not supplement any additional fruit beneath target trees. This method allowed an effective record of frugivore visitation, defined as the presence of any vertebrate frugivore underneath the target fruiting tree (i.e. irrespective of whether they feed on fruits). In contrast with Andujar, fruit removal in Matasgordas and Reserva could not be systematically quantified, although we noted all fruit removal events that were occasionally recorded by our camera-traps. Cameras were placed from 3 to 5 m distance from the focal trees and were automatically activated any time a movement occurred, taking a three-photo sequence. We set a trigger delay of 1 min after every three-photo sequence. Frugivore activity was monitored from the start of fruit ripening (mid-September) until all or most fruits have fallen (usually, from late November to early December). For a given camera and frugivore species, we considered successive visits separated by more than 5 min between them. Although 5 min might lead to recording the same individuals several times, we were interested in quantifying successive visits by the same individuals since such accumulated number of visits is likely to lead to higher fruit removal^44^. In overall, cameras in Matasgordas were working 558, 609, and 694 trap-days in 2019, 2021 and 2022, respectively. In Reserva cameras were working for a total of 420 trap-days in 2022.

For the sake of simplicity, in all three study sites recorded frugivore species were grouped into seven functional groups: ungulates, livestock, carnivores, lagomorphs, rodents, corvids, and ‘other birds’. Because the time interval for considering successive visits by a given frugivore species differed between Andujar (30 min) and Matasgordas/Reserva (5 min), across study site comparisons of the number of frugivore visits would be misleading. However, we here are particularly interested in comparing the proportion of visits by different functional groups of frugivores (e.g., ungulates *vs*. carnivores *vs*. birds) both within and between study sites, which is not subject of such limitation. Proportions were compared with Chi-squared tests using the R *stats* package^82^.

Methodological approaches for recording frugivore visits varied among study sites. In Andújar, Iberian pear fruits (n = 30) were provided beneath each target tree, while in Matasgordas and Reserva, no fruits were added. This difference likely contributed to increased frugivore visitation in Andújar compared to the other sites. Moreover, the camera-trap detection range in Andújar was narrower, focusing mainly on fruit depots, while in Matasgordas and Reserva, it covered a larger area beneath each target tree. This discrepancy might have led to fewer frugivore records in Andújar. These variations in monitoring techniques prevented a direct comparison of frugivore visit numbers across sites, prompting focus on the percentages of visits by frugivore groups. Additionally, camera-trapping duration in Matasgordas spanned three years, whereas only one year was monitored in the other sites, potentially confounding spatial and temporal patterns of frugivore visitation. Thus, when comparing the proportion of visits by different frugivore groups, initially, all sites and sampling years were considered, followed by a more conservative approach of analyzing pairs of study sites monitored in the same year. Results from both approaches were highly consistent.

## Data Availability

The datasets generated and/or analysed during the current study are available in the Figshare repository, 10.6084/m9.figshare.25205729.v1.
